# Epithelial-to-mesenchymal transition status of primary breast carcinomas and its correlation with metastatic behavior

**DOI:** 10.1007/s10549-018-05089-5

**Published:** 2019-01-04

**Authors:** C. D. Savci-Heijink, H. Halfwerk, G. K. J. Hooijer, J. Koster, H. M. Horlings, S. L. Meijer, M. J. van de Vijver

**Affiliations:** 1 Department of Pathology, Amsterdam UMC, University of Amsterdam, Meibergdreef 9, 1105 AZ Amsterdam, The Netherlands; 2 Department of Oncogenomics, Amsterdam UMC, University of Amsterdam, Meibergdreef 9, 1105 AZ Amsterdam, The Netherlands; 3 Department of Pathology, The Netherlands Cancer Institute, 1066 CX Amsterdam, The Netherlands

**Keywords:** Breast carcinoma, Chemoresistance, Chemotherapy response

## Abstract

**Background:**

Epithelial-to-mesenchymal transition (EMT) has been implicated as an important step in the development of distant metastases. We therefore wished to study EMT status of primary breast carcinomas from patients who during follow-up developed distant metastases.

**Methods:**

mRNA expression profiles of primary breast carcinoma samples (*n* = 151) from patients who developed metastatic disease were analyzed and EMT status was designated using a previously described EMT-core signature. EMT status of the primary tumor was correlated to clinicopathological characteristics, molecular subtypes, metastasis pattern, chemotherapy response and survival outcomes. In addition, using immunohistochemistry, the expression levels of several proteins implicated in EMT were studied (*CDH1, CDH2, NAT1, SNAI2, TWIST1, VIM,* and *ZEB1*) compared with the designated EMT status and survival.

**Results:**

Utilizing the 130-gene-EMT-core signature, 66.2% of the primary tumors in the current study was assessed as EMT-activated. In contrast to our expectations, analyses revealed that 84.6% of Luminal A tumors, 65.1% of Luminal B tumors, and 55.6% of HER2-like had an activated EMT status, compared to only 25% of the basal-type tumors (*p* < 0.001). EMT status was not correlated to the pattern of metastatic disease, metastasis-specific survival, and overall survival. Similarly, there was not a significant association between EMT status of the primary tumor and chemotherapy response in the metastatic setting. Immunostaining for *NAT1* and *TWIST1* correlated with the EMT status (*p* 0.003 and *p* 0.047, respectively). Multivariate analyses showed that *NAT1* and *TWIST1 staining* was significantly associated with EMT status regardless of the estrogen receptor status of the tumors (*p* values: 0.020 and 0.027, respectively).

**Conclusions:**

The EMT status of breast cancers, as defined by the presence of a core EMT gene expression signature is associated with non-basal-type tumors, but not with the pattern of distant metastasis. Of several potential immunohistochemical EMT markers, only *NAT1* and *TWIST1* expression levels were associated with the gene expression-based EMT status.

**Electronic supplementary material:**

The online version of this article (10.1007/s10549-018-05089-5) contains supplementary material, which is available to authorized users.

## Introduction

Epithelial-to-mesenchymal transition (EMT) is a complex and dynamic process that involves transdifferentiation of the cells by means of changes in the cell state. This process of epithelial–mesenchymal plasticity plays an established role in embryogenesis and early organ development [23, 30, 48]. EMT is initiated with the activation of transcription factors such as *Snail, Twist, Slug,* and *Zeb1* and is regulated by the modulation of multiple epigenetic regulatory mechanisms [43, 48]. This process results in the loss of epithelial features and acquiring mesenchymal properties such as motility, invasiveness, and resistance to apoptosis, eventually leading to colonization and metastasis formation [40]. It is thought that once colonization of the tumor cells at distant sites has occurred, these EMT-derived mesenchymal cells with stem cell-like properties go through mesenchymal-to-epithelial transition (MET) and re-gain epithelial features and continue to proliferate [3]. Along with its role in cancer metastasis, epithelial–mesenchymal plasticity is also indicated as the origin of systemic therapy resistance in breast cancer stem cells [20, 25, 56].

Several transgenic mouse models have provided evidence for the existence of tumor cells with a mesenchymal phenotype in different types of carcinomas [18, 27, 34, 41]. Using genetically engineered knock-in reporter mouse lines and fluorescence activated cell sorting, Ye et al. have isolated *Slug*^+^ and *Snail*^+^ cells in normal mammary tissue. They have shown that epithelial-to-mesenchymal transition-inducing transcription factors (EMT-TFs) *Snail, Twist*, and *Zeb1* were expressed in stromal fibroblasts surrounding the mammary ducts, whereas *Slug* was found to be expressed in basal mammary epithelial cells. Adopting a transgenic model of mammary tumor development, as tumors progressed to more undifferentiated phase(s), they have identified that the *Snail*^+^ cancer cells dissociating from epithelium acquired an elongated morphology similar to mesenchymal cells. These cells were found to have lost E-cadherin expression and activated expression of Zeb1. During the process of tumor progression, these cells were also shown to gain CK14 expression especially at the invasive edges of the organoids. With these results, the authors have demonstrated the potential role of *Snail* and subsequent EMT activation in obtaining basal features usually seen in more aggressive breast tumor types [59].

Despite the increasing interest in this dynamic process, it is still unknown what the exact role of EMT is in the development of distant metastases in human breast cancer. Several authors have suggested that the EMT state of a tumor can range from partial to full as opposed to a static event leading to gain or loss of a function [7, 19, 21, 43]. These studies have also identified that the main tumor bulk and the invasive front of the tumor differ: the invasive front being the main area for the EMT program to interact closely with the tumor microenvironment. Individual tumor cells which undergo EMT have been defined at the invasive front of the tumor and have been described as individual cells or small cell groups detaching from the main mass into the adjacent stroma [4, 19, 33, 54, 55]. The difficulty to recognize and distinguish these individual cells from the pre-existing stromal cells has contributed to the controversy of existence of clinical evidence of EMT.

Recently, a quantitative EMT scoring system based on gene expression profiling of cell lines was identified. It was shown that each cancer type had its own characteristic EMT spectrum; however, EMT status of the tumors did not correlate with poorer survival or to chemotherapy resistance [44]. A prior EMT-core signature generated by using EMT-induced human mammary epithelial cells was found to be strongly correlated to metaplastic and claudin low breast cancer, but not to other gene expression-based subtypes; and lacked to show correlation with poorer survival outcome [46].

To explore the accordance of the concept to reconcile the EMT-ness in clinical practice, we have conducted a study utilizing gene expression profiling data from primary breast cancers from a group of patients with known metastatic disease. In the current study, the association between EMT status of the primary tumors and their pattern of metastatic disease and the possibility of determining this EMT status with the help of selected routine immunohistochemical stains was investigated.

## Materials and methods

### Patient and tumor samples

This study was conducted in line with national ethical guidelines of ‘Code for Proper Secondary Use of Human Tissue’ developed by the Federation of Medical Societies (FMWV) in the Netherlands [53]. Metastatic breast cancer patients from the *Academic Medical Center* and the *Netherlands Cancer Institute* with available frozen material from their primary tumors were identified. Relevant detailed clinical information on metastatic disease including the metastasis site, timeline of the metastatic disease, and the outcome measures (metastasis-specific survival and overall survival) was collected from a group of 151 patients. The clinicopathological features of these tumors and their affiliated metastasis pattern have been reported previously [37]. For each patient, administered chemotherapy and therapy-related data including chemotherapy response for the given regimen(s) during the metastatic process were carefully recorded as previously described in a subgroup of the patients (*n* = 142) [38].

The histologic sections from the primary tumors were reviewed and additional routine staining techniques were applied to determine the hormone receptor status of the tumors [37].

### Identification and validation of EMT status

Comparing the first and the last H-E-stained sections, the samples with more than 50% tumor cells were used for the gene expression profiling experiments. The details about the RNA isolation and gene expression microarrays HumanHT-12 v4 Expression BeadChip arrays [Illumina, Inc., > 47,000 probes] have been reported previously [39]. Full information on RNA amplification, labeling, and hybridization can be also found on the Illumina website (http//www.illumina.com). Following the robust spline normalization, the data were log2 transformed and processed by ComBat (https://www.bu.edu/jlab/wp-assets/ComBat/Download_files/ComBat.R) to tailor the batch effects.

The generated data were analyzed with help of R2 (Microarray Analysis and Visualization Platform, http//r2.amc.nl). Molecular subtypes were assessed for each tumor using the Pam50 classifier [32]. Also the percentage of tumor cells on the slides used for gene expression profiling experiments were correlated to the molecular subtypes.

To designate the EMT status of each tumor, the EMT-core gene list of Groger et al. was utilized [15]. The 130 genes of this EMT-core gene list were first mapped to the Illumina platform via Gene Symbol ID. In case of existence of multiple probes for one gene, the one with highest average signal across the samples was selected. Subsequently, a K-means clustering method was applied to separate the tumors into two groups as EMT-activated or not-EMT-activated. By means of K-means clustering method, the EMT-core gene list was also applied to an independent set composed of a subset of a combined database including 376 breast cancer samples with distant organ metastases [17]. Additionally, based on gene expression levels, *z* scores were calculated for each tumor to define the EMT status in accordance with distribution of the *z* scores. EMT status identified by K-means method and z-scores of the tumors were subsequently compared to verify the identified EMT status of the given tumor. Given the significant concordance in assigned EMT status with K-means method and the *z* scores method (*p* 3.92e-20) in our dataset, further analyses and comparisons were carried out on the EMT status based on K-means method.

To further reconcile the EMT-ness with the help of immunohistochemical stains, a subset of 46 tumors (EMT-activated, *n* = 23 and not-EMT-activated, *n* = 23) were selected. The heat map created by supervised clustering with the EMT-core signature was carefully observed. Based on the current literature information on their established role in EMT, a subgroup of 7 proteins (*CDH1, CDH2, NAT1, SNAI2, TWIST1, VIM*, and *ZEB1*) was selected for further evaluation. To test the representativeness of this subset of genes, respectively, a K-means clustering method and a *t* test were carried out first to classify the tumors into two groups according to their EMT status and then to validate the performance of this classification in the same study set (used for immunophenotypic evaluation).

#### Immunophenotypic evaluation

Whole-mount slides of the tumors from a subgroup of patients (total *n* = 46; EMT-activated *n* = 23, not-EMT-activated *n* = 23) were selected for the additional immunophenotypic evaluations.

Immunohistochemical staining for *E-Cadherin* (*CDH1*, clone 24E10, Cell signaling), *N-cadherin* (*CDH2*, clone 32N/Cadherin, BD transduction Laboratories), *NAT1* (Abcam), *SNAI2* (Abcam), *Vimentin* (clone D21H3, Cell Signaling), and *ZEB1* (Sigma Life science) was performed using an automated slide preparation system (Benchmark XT, Ventana Medical Systems, Tucson Arizona, USA). The signal detection for immunohistochemistry was performed with a biotin-free ultraview universal DAB detection Kit (Ventana medical systems). Immunohistochemical staining for *TWIST1* (Clone Twist2C1a, Abcam) was performed manually with a Bright DAB detection.

The immunostained slides were scored by two pathologists (C.D. S-H, S.L.M). The invasive edges and the main tumor mass were evaluated separately. Immunostaining patterns for the other antibodies were evaluated semi-quantitatively; the extent and the intensity of the expression were scored for each antibody. For *NAT1, SNAI2, TWIST,* and *ZEB1*, a cut-off value of 10% was used to designate tumors as positive or negative. Absence of E-Cadherin expression in > 1% of the tumor cells was considered as loss of expression and presence of *N-cadherin* and *Vimentin* expression in > 1% of tumor cells was noted as gain of expression. Immunohistochemical findings were then correlated to the designated EMT status.

### Statistical analysis

The association between the EMT status of the tumors and clinical variables was further investigated with multivariate logistic regression tests applying SPSS Statistics for Windows (Release version 21.0; IBM Corp. 2012, Armond, NY). Univariate and additional multivariate regression tests [adjusted for ER (positive *versus* negative)] were utilized for the comparisons of the immunostaining results to the EMT status. Hazard ratios (HRs) were presented with their 95% confidence intervals (CIs). The statistical tests were two-sided and *p* value being less than 0.05 was considered to be significant.

## Results

We studied epithelial–mesenchymal transition status, as assessed by gene expression profiling, for 151 primary invasive breast carcinomas of patients whom all developed metastatic disease. Clinical and pathologic characteristics of the primary breast tumors have been previously described [37] and are shown in Table 1.

**Table 1:** Clinical and pathological characteristics of metastatic breast cancer patients

	*N*	%
Age at diagnosis, years		
< 50	83	52.9
> 50	74	47.1
Surgical procedure		
None	4	2.8
Mastectomy	73	51.8
Breast conserving	64	45.4
Adjuvant therapy		
None	30	21.1
Only CT	50	35.2
Only HT	17	12.0
CT + HT	45	31.7
Lymph node status		
None	43	29.3
1–3 positive	48	32.7
> 3 positive	56	38.1
Histology		
Ductal	134	86.5
Lobular	14	9.0
Other	7	4.5
Tumor grade		
1	13	8.6
2	84	55.3
3	55	36.2
Time to distant metastasis^a^		
Early	117	77.0
Late	35	23.0
Metastasis at first presentation		
No	141	92.8
Yes	11	7.2
Multiple metastasis sites at first presentation		
No	97	64.2
Yes	54	35.8
Multiple metastasis sites during follow-up		
No	37	24.5
Yes	114	75.5

*CT* chemotherapy, *HT* hormonal therapy

^a^Cut-off point 5 years

**Fig. 1:**
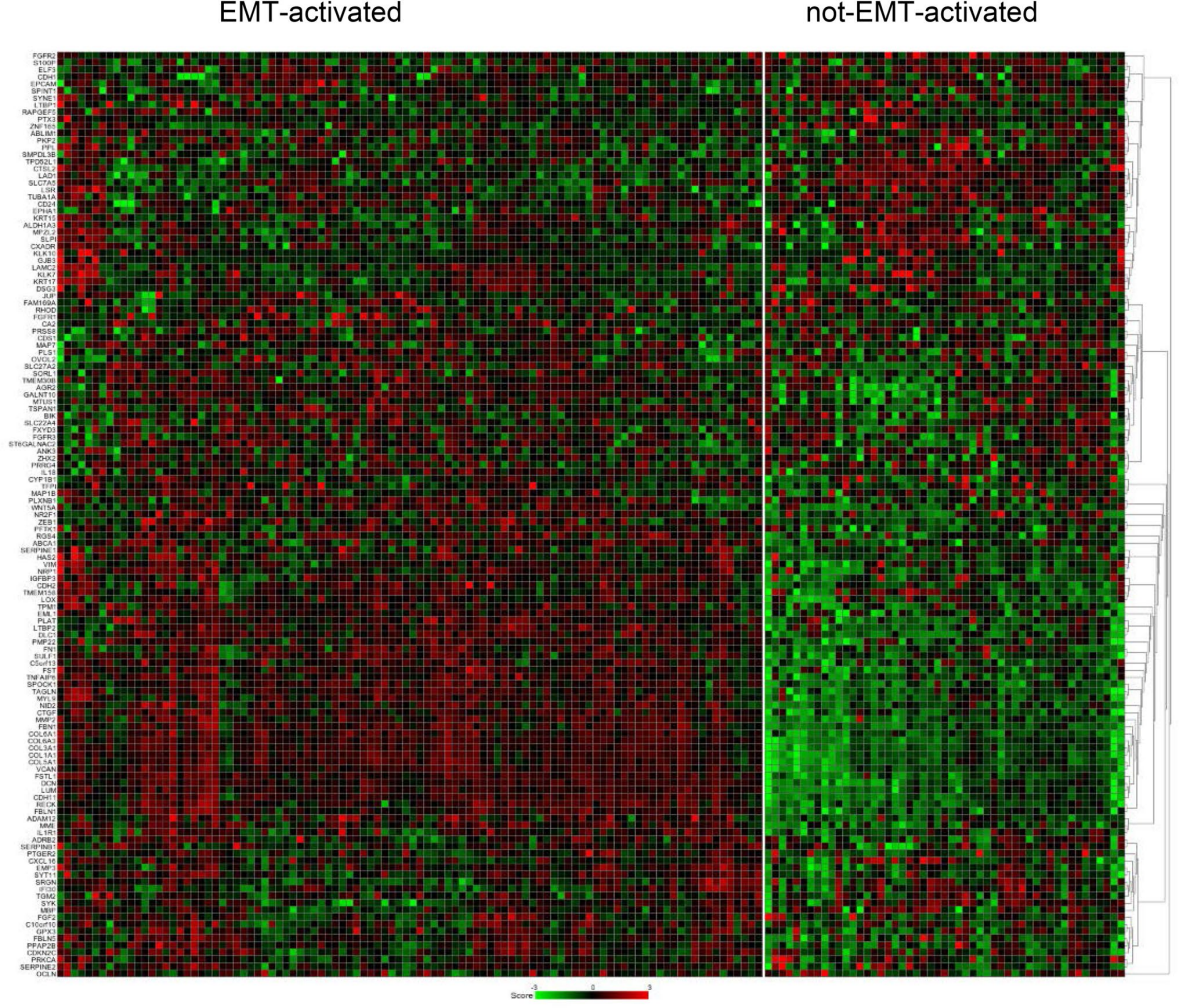
Heat map shows the gene expression profiling pattern of 130-genes of EMT-core signature among 151 patients. For each primary tumor, the expression level of the specific gene is exhibited as red, if up-regulated and green, if down-regulated

Using the 130-gene-EMT-core signature, 66.2% (*n* = 100) of the primary tumors in the current study was assigned as EMT-activated and 33.8% (*n* = 51) as not-EMT-activated. The heat map shown in Fig. 1 displays the gene expression profiling pattern of 130 genes of the EMT-core signature. In the independent dataset, 72.9% (*n* = 274) of the tumors was identified as EMT-activated and 27.1% (*n* = 102) as not-EMT-activated.

Comparisons of the histopathological characteristics of the tumors from EMT-activated group with the tumors from not-EMT-activated group showed that 76.9% of Grade 1 and 78.5% of grade 2 tumors had an activated EMT status as opposed to the 45.5% of grade 3 tumors (*p* < 0.001). Histologic type or the size of the tumor was not found to be correlated with the gene expression-based EMT status (*p* 0.635).

Among ER-positive and PR-positive tumors 77.8% and 81.1% were identified as EMT-activated, respectively (*p* < 0.001). Of Luminal A tumors 84.6%, of Luminal B tumors 65.1%, and of HER2-like 55.6% was assessed as having an activated EMT status, whereas only 25% of the basal-type tumors were assigned to the EMT-activated group (*p* < 0.001). Similarly, in the independent dataset [17] of total 376 tumors, 100% of the Luminal A tumors, 97.5% of the Luminal B tumors, 92,1% of HER2-like tumors, and 8,3% of the basal-type tumors were designated as EMT-active (*p* < 0.001). Mean tumor percentage in the basal-type tumors was 72.1% (range 66.8–77.3). In the non-basal tumor groups, mean percentage was 58.9% in luminal A type, 65.8% in luminal B type, and 62.1% in HER2-like tumors (*p* < 0.001).

EMT status did not differ between the patients who developed metastasis within 5 years’ time and the ones who developed metastatic disease later than 5 years (*p* 0.310). Regarding the metastasis site, out of 108 patients who developed bone metastasis 72.2% had an EMT-activated primary tumor (*p* 0.021) versus 65.2% of the tumors of the patients with visceral metastasis (*p* 0.698).

**Fig. 2:**
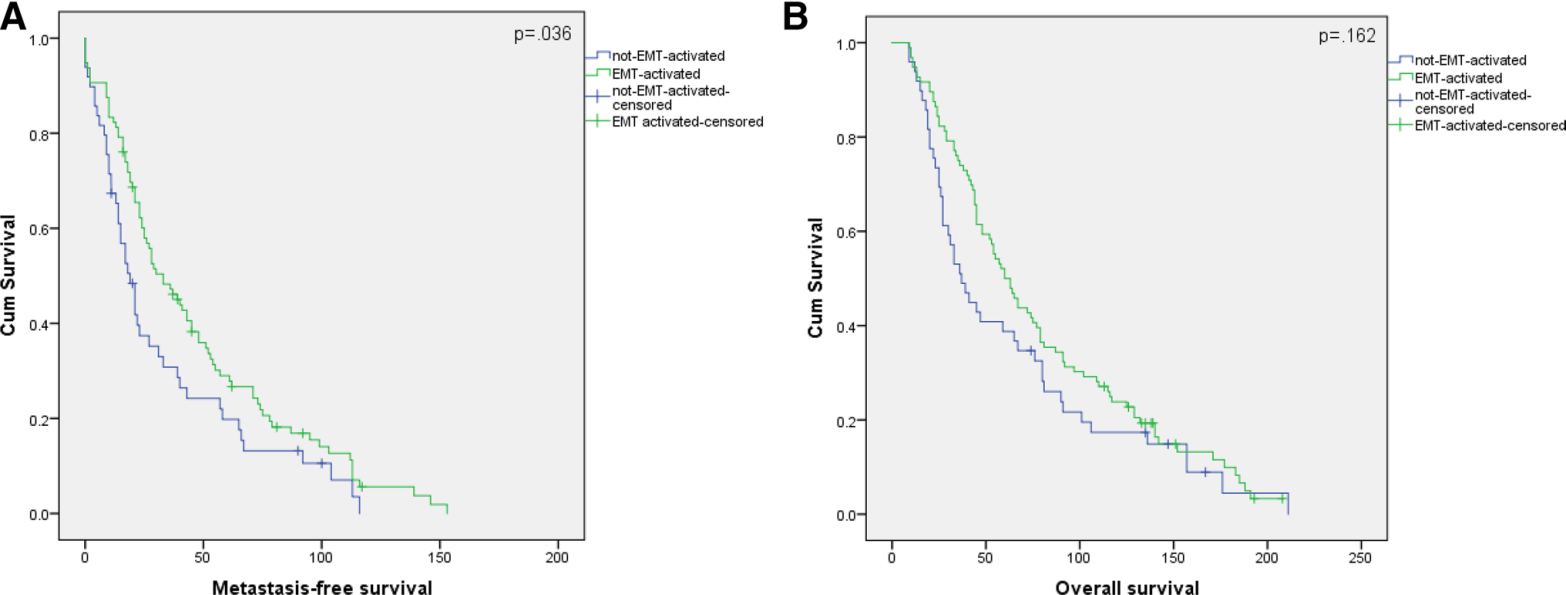
Metastasis-specific (**A**) and overall (**B**) survival curves of breast cancer patients according to EMT status. Kaplan–Meier plots of patients show that EMT-activated tumors had better survival outcomes compared to the tumors with not-EMT-activated status

Median overall survival time was 60 months and 37 months for the EMT-activated and the not-EMT-activated group, respectively (*p* 0.162). Metastasis-specific survival time was 33 months for the patients with EMT-activated tumors and 19 months for those with non-EMT-activated tumors (*p* 0.036) (Fig. 2).

118 patients underwent chemotherapy treatment; 48.1% of the patients with EMT-activated tumors showed a response versus 31.4% of those with not-EMT-activated ones (*p* 0.130).

We subsequently wished to study the correlation of the gene expression-based EMT status of the tumors with the expression of EMT-associated proteins in using immunohistochemistry in a subset of 46 tumors (23 EMT-activated; 23 not-EMT-activated); the findings are summarized in Table 2.

**Table 2:** Correlation of immunohistochemical findings and EMT status

	IHC	EMT status		
		Non-activated	Activated	*p*
CDH1	Negative	3	2	1.000
	Positive	20	21	
CDH2	Negative	20	18	0.699
	Positive	3	5	
NAT1	Negative	16	5	0.003
	Positive	7	18	
SNAI2	Negative	7	6	1.000
	Positive	16	17	
TWIST1	Negative	20	13	0.047
	Positive	3	10	
VIM	Negative	19	23	0.109
	Positive	4	0	
ZEB1	Negative	17	10	0.071
	Positive	6	13	

*IHC* immunohistochemistry, *EMT* epithelial-to-mesenchymal transition

Immunohistochemical evaluation for all stains revealed comparable expression patterns at the invasive edges and at the center of the tumors; therefore, analyses were further carried out based on a single score.

The expression patterns of staining for *E-cadherin, N-cadherin, Vimentin, SNAI2,* and ZEB1 did not differ between the EMT-activated and not-EMT-activated groups (*p* values: 1.000, 0.699, 0.109, 1.000, and 0.071, respectively).

*NAT1* expression was scored as positive in 25 cases with a range of 30–100% positivity in the tumor cells. Out of positively stained cases, 72% was assigned to the EMT-activated group; of negatively stained cases 76.2% was marked as not-EMT-activated (*p* 0.003). Further evaluations showed that NAT1 expression was not significantly correlated to the overall survival (*p* 0.223) and metastasis-specific survival (*p* 0.146).

**Fig. 3:**
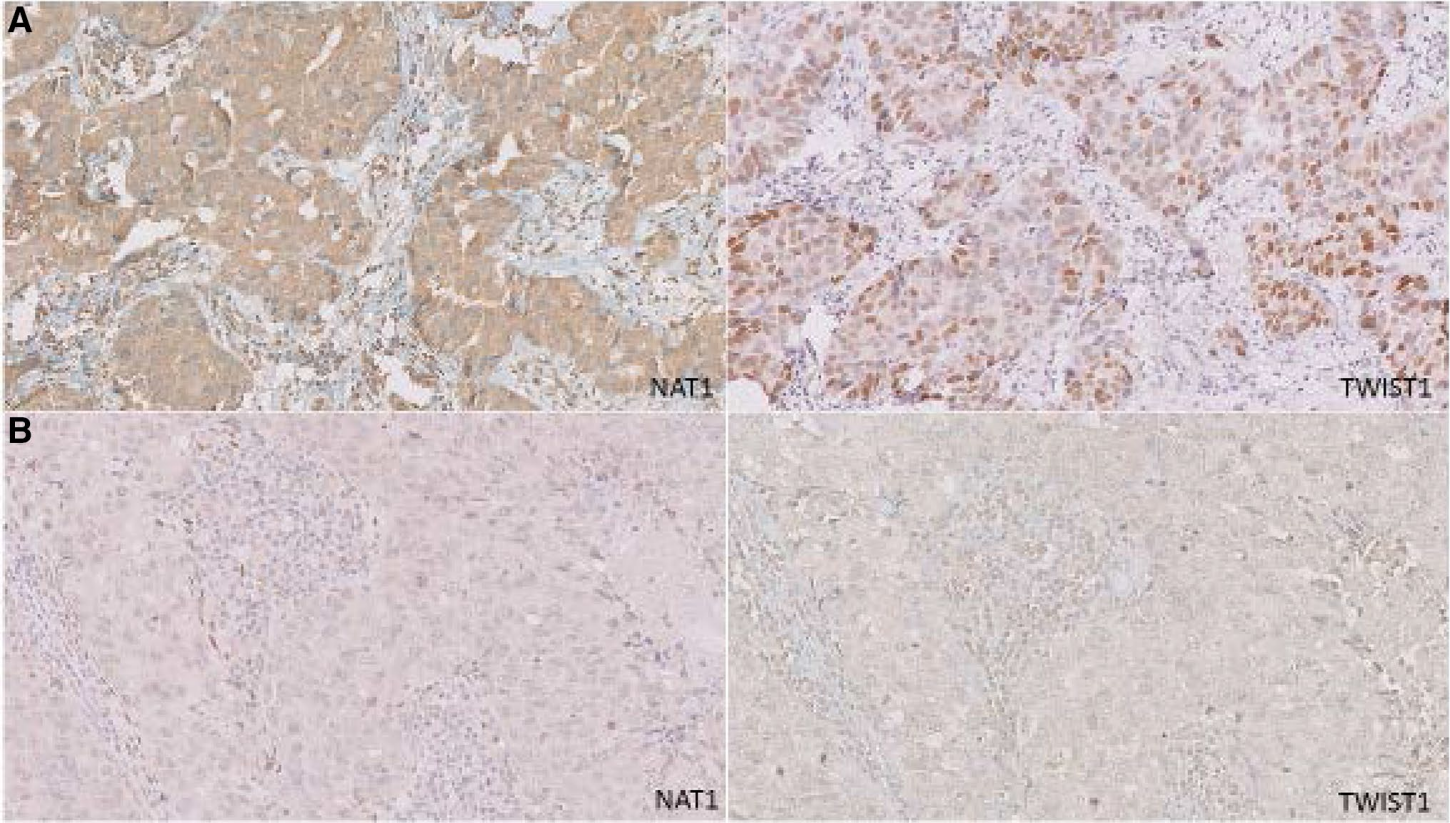
Microscopic images displaying immunoexpression for NAT1 and TWIST1 for an EMT-activated (**A**) and a not-EMT-activated (**B**) tumor

*TWIST1* expression was found to be positive in 13 cases with 76.9% of these tumors being EMT-activated and of 60.6% of *TWIST1* negative tumors belonged to the not-EMT-activated group (*p* 0.047). *TWIST1* expression was not found to be significantly correlated with overall survival and metastatic-specific survival (*p* 0.675, *p* 0.461, respectively). Figure 3A, B illustrates expression of NAT1 and TWIST1 in an EMT-activated and a not-EMT-activated tumor.

To investigate the additional role of *NAT1* and *TWIST1* staining to predict EMT status, multivariate regression analyses were applied. Multivariate analyses results are displayed in Table 3 and shows that positive *NAT1* and *TWIST1* staining was significantly correlated to EMT-activated status independent of ER status of the tumor (*p* values: 0.020 and 0.027, respectively).

**Table 3:** Multivariate analyses results displaying the correlation between immunohistochemical findings and EMT status

	B	Wald *x*^2^	*p*	Odds ratio	95% CI
ER status	− 1.11	0.68	0.410	0.33	0.02–4.63
NAT1 status	3.18	5.37	0.020	23.98	1.63–352.24
TWIST1 status	2.12	4.92	0.027	8.35	1.28–54.55

*EMT* epithelial-to-mesenchymal transition, *ER* estrogen receptor

## Discussion

In this study, gene expression profiles from primary breast carcinomas of patients with known metastatic disease have been utilized to assess the EMT status of the primary tumor. Subsequently, the designated EMT status has been correlated to the metastatic behavior and survival outcomes. In addition, the expression of EMT-associated proteins to the EMT status as assessed by gene expression profiling was studied.

The previously suggested reciprocal link between basal-type breast cancer as assessed by immunohistochemistry and EMT [35] was not found in our dataset. Unexpectedly, the low-grade tumors tended to be more frequently EMT-activated than high-grade tumors.

The role of epithelial-to-mesenchymal transition (EMT) in cancer progression has been demonstrated in several tumor models. Yet, the translation of this concept to clinical breast cancer remains problematic and it has been argued that EMT may not be required for the development of distant metastases [45]. Recent investigations focusing on these debates have led to adoption of a new concept of EMT indicating the flexibility and intermediate hybrid state of this process rather than a rigid state [1, 19, 21, 43, 60]. These recently proposed transitional states and the heterogeneity of EMT may explain the difficulty to visualize the EMT status. In our study, we were not able to show any significant association between EMT status and the metastasis time (early versus late metastasis). Overall survival and metastasis-specific survival outcomes did not differ significantly between EMT-activated and not-EMT-activated group, either. Although these results seem to be opposing to common concept that EMT-active status has bad prognostic implications, they can be due to proposed intermediate hybrid states. Tan et al. have already addressed this issue with their study including several types of cancer tissue [44]. Applying a generic EMT signature, they have quantitatively estimated the extent of EMT in human tumor samples and cell lines. In this study, authors have not found a relation between EMT status and overall and disease-free survival. Particularly in the breast carcinoma samples, they have shown that tumors with mesenchymal (*Mes*) profile appeared to have better prognosis than the ones with epithelial (*Epi*) profile. The authors have suggested the role of stromal component and the distribution of molecular subtypes for the contradictory results. Concordantly, in this study, we have demonstrated that the percentage of tumor cells, hence the epithelial component differed among the molecular subtypes and luminal type tumors, had relatively more stromal component than the basal-type tumors, which may explain the association between luminal type tumors and the EMT-core signature (*p* < 0.001).

Next to its association with cancer progression and metastasis formation, EMT has been linked to chemoresistance in several cancer types [8, 12, 20, 40, 56, 62, 64]. Several studies have demonstrated that cells with an EMT profile, rather than directly establishing metastasis, showed more resistance to chemotherapy (CT) and have indicated the potential role of EMT-targeted therapy. In our current study, we were not able to demonstrate a link between EMT status of the primary breast tumors and response to CT in the metastatic setting. The study conducted by the group of Tan has also failed to show a direct translation of EMT status to chemotherapy resistance [44]. These authors have concluded that in addition to acquiring EMT, gaining stem cell-like properties plays an important role in chemoresistance. Several studies have already shown that overexpression of EMT-inducing transcription factors leads to changing luminal lineage cells to a more stem cell-like trait suggesting that these breast cancer stem cells showing an EMT-like profile are more chemotherapy resistant [8, 12, 16, 40]. A generic EMT signature which is developed to assess the EMT status, may not be the optimal tool to assess the stemness of the cancer cells and their potential response profile [3, 13, 35, 44].

Activation of an EMT program has been suggested as a critical event for cancer progression which grants epithelial cancer cells with more invasive mesenchymal phenotypes [48]. Direct visualization of these cells going through this process and their morphological changes remains an area of interest. To recognize the cancer cells with EMT phenotype, we have performed immunostaining for *CDH1, CDH2, NAT1, SNAI2, TWIST1, VIM*, and *ZEB1*. We were not able demonstrate the significant difference between EMT-activated and not-EMT-activated group regarding *CDH1, CDH2, SNAI2, VIM*, and *ZEB1* expression, in the tumor bulk as well as at the invasive edges of the tumor tissue. Noteworthy, the staining pattern of *TWIST1* and *NAT1* have appeared to be related to the EMT status of the primary tumor. We have already pointed out the link between *NAT1* and EMT status in a previous gene expression profiling-based study [39] and its potential role, particularly as a drug target in cancer development [11, 42, 49]. N-acetyltransferase 1 (*NAT1*) is a human enzyme that metabolizes arylamine and hydrazine-class drugs [5]. Next to its association with survival of breast epithelial cells [2], *NAT1* has been proposed as a useful biomarker for breast cancer [11, 22, 50, 51]. Several studies have shown that knockout of *NAT1* led to the modification of intracellular actin which resulted in suppression of invasion and metastasis. Loss of NAT1 has also been reported to reduce the cell-to-cell contact growth and enhance the up-regulation of E-cadherin [50, 51]. TWIST1, a helix-loop-helix domain containing transcription factor, carries a key regulator role in organogenesis and its hypermethylation and overexpression have been identified in several tumor types [10, 14, 26, 28, 31, 57, 63]. This transcription factor has been implicated in many steps of cancer progression (invasion, metastasis, angiogenesis, cancer stemness) [6, 9, 52, 58]. The association between TWIST1 and EMT, which was first proposed by Yang et al. [57], has been confirmed in different types of cancer [24, 29, 36, 47, 61].

Many investigators have faced difficulties to detect cancer cells with EMT phenotype. To overcome the main obstacle which is to differentiate the stromal fibroblasts from the cells with EMT phenotype, Yu et al. conducted a study using RNA in situ hybridizations on HER2-positive breast tumors in order to distinguish primary tumor cells from the surrounding stromal cells [60]. By using dual-colorimetric RNA in situ hybridizations, they were able to identify breast cancer cells co-expressing epithelial and mesenchymal markers. Contrary to expectations, these biphenotypic cells were observed mainly in draining lymph nodes but not at the invasive fronts of primary tumors. Alongside the heterogeneous nature of EMT process and possibility of an incomplete EMT state, it has also been suggested that molecular alterations that initiate a signal transduction cascade leading to EMT properties does not necessarily prompt acquirement of a complete mesenchymal phenotype [7].

In conclusion, our results fails to draw a direct line between the gene expression-based EMT status of a primary tumor and its associated metastatic behavior. Our findings suggest that the EMT status of the tumor, defined by the EMT-core signature, may largely be the result of the amount of stroma in the tumor (which is often larger in grade 1 tumors compared to grade 3 tumors). In this study, we have also demonstrated that immunostaining for *NAT1* and *TWIST1* may be of help to identify the tumor cells with EMT phenotype. We believe that our study is a valuable addition to the current literature and gives additional perspective on EMT in human metastatic breast carcinomas.

## Electronic supplementary material

Below is the link to the electronic supplementary material.

Supplementary material 1 (XLSX 170462 KB)

Supplementary material 2 (DOCX 69 KB)

## Abbreviations

EMT: Epithelial-to-mesenchymal transition
CT: Chemotherapy
ER: Estrogen receptor
HER2: Human epidermal growth factor receptor 2
