# Supplementary figures and images for: Epithelial-to-mesenchymal transition status of primary breast carcinomas and its correlation with metastatic behavior
Source: Breast Cancer Res Treat. 2019 Jan 4;174(3):649–59. doi: 10.1007/s10549-018-05089-5 (PMC6438946; doi:10.1007/s10549-018-05089-5)

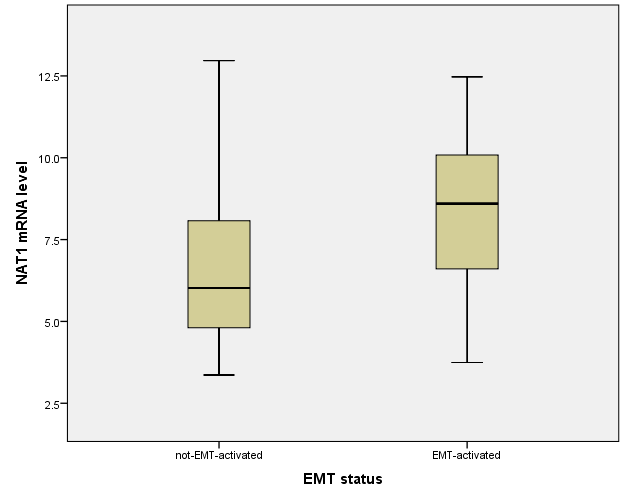

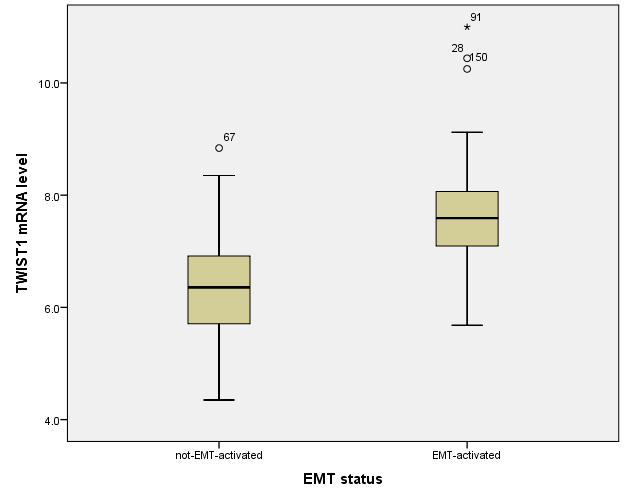


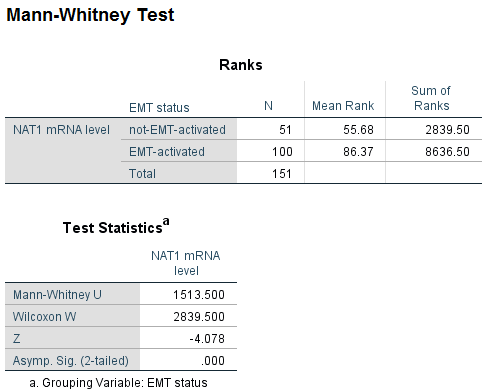

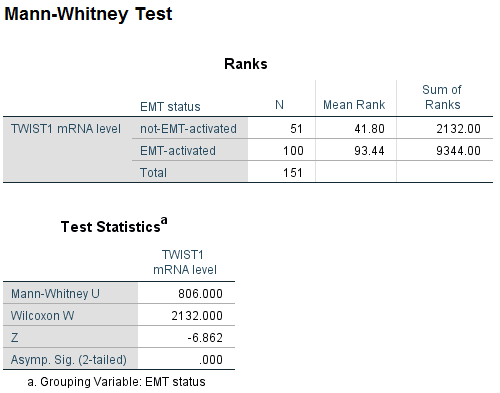

Supplement: Supplementary file 2 — Supplementary material 2 (DOCX 69 KB) [file 10549_2018_5089_MOESM2_ESM.docx]
